# Association of Serum 25-Hydroxyvitamin D with Lifestyle Factors and Metabolic and Cardiovascular Disease Markers: Population-Based Cross-Sectional Study (FIN-D2D)

**DOI:** 10.1371/journal.pone.0100235

**Published:** 2014-07-07

**Authors:** Maija E. Miettinen, Leena Kinnunen, Jaana Leiviskä, Sirkka Keinänen-Kiukaanniemi, Eeva Korpi-Hyövälti, Leo Niskanen, Heikki Oksa, Timo Saaristo, Jaakko Tuomilehto, Mauno Vanhala, Matti Uusitupa, Markku Peltonen

**Affiliations:** 1 Diabetes Prevention Unit, National Institute for Health and Welfare, Helsinki, Finland; 2 Disease Risk Unit, National Institute for Health and Welfare, Helsinki, Finland; 3 Institute of Health Sciences, University of Oulu, Oulu, Finland; 4 Unit of Primary Health Care, Oulu University Hospital, Oulu, Finland; 5 Department of Internal Medicine, South Ostrobothnia Central Hospital, Seinäjoki, Finland; 6 Finnish Medicines Agency, Helsinki, Finland; 7 Faculty of Health Sciences, University of Eastern Finland, Kuopio, Finland; 8 Tampere University Hospital, Tampere, Finland; 9 Pirkanmaa Hospital District, Tampere, Finland; 10 Finnish Diabetes Association, Tampere, Finland; 11 Center for Vascular Prevention, Danube-University Krems, Krems, Austria; 12 King Abdulaziz University, Jeddah, Saudi Arabia; 13 Primary Health Care Unit, Central Finland Central Hospital, Jyväskylä, Finland; 14 Institute of Public Health and Clinical Nutrition, University of Eastern Finland, Kuopio, Finland; 15 Research Unit, Kuopio University Hospital, Kuopio, Finland; 16 Department of Chronic Disease Prevention, National Institute for Health and Welfare, Helsinki, Finland; University of Oxford, United Kingdom

## Abstract

**Objectives:**

Low serum 25-hydroxyvitamin D (25OHD) level has been associated with an increased risk of several chronic diseases. Our aim was to determine lifestyle and clinical factors that are associated with 25OHD level and to investigate connection of 25OHD level with metabolic and cardiovascular disease markers.

**Design:**

In total, 2868 Finnish men and women aged 45–74 years participated in FIN-D2D population-based health survey in 2007. Participants that had a serum sample available (98.4%; n = 2822) were included in this study. 25OHD was measured with chemiluminescent microparticle immunoassay method.

**Results:**

The mean 25OHD level was 58.2 nmol/l in men (n = 1348) and 57.1 nmol/l in women (n = 1474). Mean 25OHD level was lower in the younger age groups than in the older ones (p<0.0001 both in men and women). This study confirmed that low physical activity (p<0.0001 both in men and women), smoking (p = 0.0002 in men and p = 0.03 in women) and high BMI (p<0.0001 in women) are factors that independently associate with low 25OHD level. Of the metabolic and cardiovascular disease markers high triglyceride concentration (p = 0.02 in men and p = 0.001 in women) and high apolipoprotein B/apolipoprotein A1 ratio (p = 0.04 in men and p = 0.03 in women) were independently associated with low 25OHD level.

**Conclusions:**

Higher age did not predict lower 25OHD level in this study population of aged 45–74 years which may derive from a healthy life-style of “active pensioners”. Low physical activity and smoking came up as independent lifestyle factors associated with low 25OHD level. Defining the molecular mechanisms behind the associations of 25OHD with low physical activity and smoking are important objective in future studies. The association of 25OHD with BMI, high triglyceride concentration and apolipoprotein B/apolipoprotein A1 ratio may be related to the role of vitamin D in inflammation, but more detailed studies are needed.

## Introduction

Low serum 25-hydroxyvitamin D [25OHD] level has been associated with several diseases including cancer, type 2 diabetes, osteoporosis, autoimmune diseases, cardiovascular diseases and schizophrenia [1].

Vitamin D is naturally synthesized from 7-dehydrocholesterol by the skin's exposure to ultraviolet B radiation. The best estimate of an individual's vitamin D status is the measurement of circulating vitamin D, 25OHD [1], which provides an indication of vitamin D stores obtained from both UV-light and dietary intake. Dietary sources of Vitamin D include fatty fish and fortified foods like milk and margarine. At the time of sample collection of the present study (year 2007) fish was the most important dietary source of vitamin D for both sexes among Finns aged 25–64 years [2]. Vitamin D is first hydroxylated in the liver to 25OHD and again in the kidney to form the biologically active 1,25-dihydroxyvitamin D. Low 25OHD level may be a result of inadequate sun exposure, deficient consumption of vitamin D-rich products or malabsorption of vitamin D [3].

25OHD concentration of 25 nmol/l has traditionally been considered as a boundary for vitamin D deficiency [4], [5]. However, in recent years several experts have suggested a cut-off level of 50 nmol/l for vitamin D deficiency [1], [6]. Concentration of 75 nmol/l or higher has been suggested as an optimal 25OHD level [6]. However, a reverse J-shaped association between 25OHD level and all-cause mortality has been suggested in the NHANES study [7]. The nadir of risk was shown to be 81 nmol/l [7]. In traditionally living indigenous populations in East Africa the mean 25OHD level in adult population has been shown to be 115 nmol/l [8]. There is no consensus about the toxic 25OHD concentration, but 375–500 nmol/l and higher than 600 nmol/l have been suggested [9], [10].

A combination of increased triglyceride-rich very low-density lipoproteins and small, dense low-density lipoproteins (LDL) and decreased high-density lipoproteins (HDL) is a common feature in metabolic syndrome (MetS) [11], [12]. Apolipoprotein A1 (apoA1) is the main protein in HDL particles and apolipoprotein B (apoB) in all the other lipoproteins [13], [14]. ApoB/apoA1 ratio combines the atherogenic and antiatherogenic lipoproteins and an elevated apoB/apoA1 ratio has been connected to MetS [15]. ApoB/apoA1 ratio has also been shown to be a good predictor of cardiovascular disease and atherosclerosis [15]–[19], and it has been suggested that ApoB/apoA1 ratio should be used instead of cholesterol concentrations as a risk marker of myocardial infarction [17]. Several studies have found an association between low 25OHD level and MetS [20], [21], but little is known of the link between 25OHD level and different components of the lipid disorders related to MetS, like elevated triglyceride and apoB concentrations and an increased apoB/apoA1 ratio.

Our aim was to determine the lifestyle and clinical factors that are associated with 25OHD level and to investigate connection of 25OHD level with metabolic and cardiovascular disease markers. We found several factors associated with 25OHD level that should be considered when investigating the role of vitamin D in different diseases.

## Materials and Methods

### Subjects

This study is a part of the FIN-D2D health survey 2007, which is a joint project within the Finnish National Diabetes Prevention program FIN-D2D [22], [23]. Initially, a random sample of 4500 people aged 45–74 years, stratified according to gender, 10-year age groups (45–54, 55–64, and 65–74 years), and geographical areas (Hospital Districts of Pirkanmaa, Central Finland and South Ostrobothnia), was selected from the National Population Register in August 2007. Sample size of the population where this cohort was extracted in 2007 was 349326. The study participants were invited by mail to a health examination. The participants attended a health examination carried out by a trained nurse according to the multinational monitoring of trends and determinants in cardiovascular disease (MONICA) protocol. A total of 2868 persons (64%) participated in the health examination during October, November and December in 2007. Of these persons those that had a serum sample available were included in the present study (98.4%; n = 2822). All participants signed an informed consent form. Ethical committee of the Hospital District of Helsinki and the Uusimaa approved the study.

Waist circumference, triglycerides, HDL cholesterol, blood pressure and fasting plasma glucose were used to define the presence of metabolic syndrome according to the International Diabetes Federation 2009 criteria [24]. Information on glucose tolerance status during 2 hour oral glucose tolerance test together with reported drug-treated type 2 diabetes was used to indicate presence of normal glucose regulation (NGR), impaired glucose tolerance (IGT), impaired fasting glucose (IFG), screen-detected type 2 diabetes (SDM) or type 2 diabetes according to the WHO 1999 diagnostic criteria [25]. Participant's cardiovascular risk was estimated with the Framingham [26] risk score, and with the apoB/apoA1 ratio.

### Methods

Height was measured to the nearest 0.1 cm. Weight was measured in light clothing. Body mass index (BMI) was calculated as weight (kg) divided by the square of height (m). Waist circumference was measured midway between the lower rib margin and the iliac crest. Serum triglycerides, HDL cholesterol, and plasma glucose were measured by enzymatic methods with Abbott Architect analyzer (Abbott Laboratories, Abbott Park, IL, USA). ApoA1 and apoB were measured with immunoturbidimetric methods and high sensitivity C-reactive protein (hs-CRP) was measured with ultrasensitive immunoturbidimetric method using Abbott Architect reagents. The inter-assay coefficients of variation (CV) for triglycerides, HDL cholesterol, and glucose were 1.2%, 2.5%, and 1.4%, respectively. CVs for apoA1, apoB, and hs-CRP were 1.3%, 2.5% and 2.9%, respectively.

Data of education and total household income were used as indicators of the socio-economic status. Educational level was divided into groups according to years of completed education: low (0–9 years), medium (10–12 years) and high education (13 years or more). Total household income per year was reported in three categories: low (<30 000 €/year), medium (30 001–60 000 €/year) and high (60 001–80 000 or more €/year).

### Vitamin D analyses

The serum samples were collected during the end of year 2007 within three months (October–December). The samples were stored frozen in −20°C until analysis (4 years). 25OHD was determined by chemiluminescent microparticle immunoassay by Architect *i* system (Abbott Laboratories, Abbott Park, IL, USA). The interassay CVs of 25OHD were 6.7% and 3.8% at the levels of 34 nmol/l and 102 nmol/l, respectively. The bias compared to all-laboratory trimmed mean in the Vitamin D International External Quality Assessment Scheme (DEQAS) was −6.4%±7.1 (mean ± SD). 25OHD concentration lower than50 nmol/l was considered as vitamin D deficiency, between 50 nmol/l and 75 nmol/l as vitamin D sufficiency and higher than 75 nmol/l as an optimal 25OHD level.

### Statistical analyses

Descriptive statistics (means, standard deviations and proportions) were used to describe the study population. Age- and gender-specific 25OHD levels were calculated among the participants by lifestyle factors and cardiovascular disease markers. In these analyses, continuous variables were categorized in order to evaluate linearity in associations. Univariate comparisons between groups were evaluated by the chi-square test or logistic regression models. Analysis of covariance (ANCOVA) and logistic regression models were further used to evaluate the associations between 25OHD concentrations and lifestyle factors and metabolic disease markers adjusting for possible confounding factors. The possible confounding factors were pre-determined and included age, gender, BMI, month of sample collection, physical activity and smoking. Assumptions of the ANCOVA models were evaluated by graphical methods. The goodness of fit in logistic regression models was assessed with the Hosmer-Lemeshow test. No interaction terms were included in the models. Statistical analyses were carried out using the Stata statistical package 10.1 (Stata-Corp. 2007. Stata Statistical Software: Release 10.1. College Station, TX; StataCorp LP).

## Results

Characteristics of the study subjects are shown in table 1. Information on age, month of sample collection, use of vitamin D supplements, HDL concentration, triglyceride concentration, fasting glucose, hs-CRP and apoB/apoA1 ratio (men) was available from 100% of the participants. Information on physical activity, smoking, education, waist circumference, blood pressure, Framingham score, and apoB/apoA1 ratio (women) was available from >95% of the participants. Information on household income and on use of hormone replacement therapy was available from >90% of the participants. The main reason for the missing information was that the participants did not answer all questions in the questionnaire included in the study.

**Table 1:** Characteristics of the study subjects of the FIN-D2D study (n = 2822).

	Men		Women		p
	Mean	SD	Mean	SD	
n (%)	1348 (47.8)		1474 (52.2)		
Age (years)	60.2	8.5	59.4	8.3	0.005
BMI (kg/m^2^)	27.5	4.2	27.5	5.3	0.61
Use of vitamin D supplements (%)	18.0		37.0		<0.001
Smokers (%)	19.6		11.7		<0.001
Use of hormone replacement therapy (%)			24.8		
Menstruation			21.4		
Metabolic syndrome (%)	59.8		54.0		0.002
Waist circumference (cm)	100.0	11.8	90.4	13.3	<0.001
HDL cholesterol concentration (mmol/l)	1.32	0.32	1.55	0.34	<0.001
Triglyceride concentration (mmol/l)	1.49	0.99	1.28	0.61	<0.001
Diastolic blood pressure	83.0	9.9	80.4	9.0	<0.001
Systolic blood pressure	138.4	18.7	135.4	18.8	<0.001
Fasting glucose (mmol/l)	6.4	1.3	6.0	1.1	<0.001
Type 2 diabetes (%)	20.8		13.3		<0.001
hs-CRP	2.6	8.0	2.9	7.6	0.32

### 25OHD level and its relation to lifestyle and related factors

The results of the univariate analysis of the 25OHD level in men and women are presented in Table 2. Higher 25OHD level was associated with higher age (men p<0.0001; women p<0.0001). Month of sample collection was associated with 25OHD, the mean level being approximately 8% higher in samples collected during October than during November and December (men p<0.0001; women p = 0.001). Higher BMI was associated with lower 25OHD level (men p = 0.04; women p<0.0001). Only 1.5% (n = 20) of the men and 3.1% (n = 46) of the women had BMI lower than 20 and thus a meaningful analysis of this group separately was not possible. Low physical activity was associated with lower 25OHD level (men p<0.0001; women p<0.0001). Participants that did not use vitamin containing supplements had lower 25OHD level than those using supplements (men p<0.0001; women p<0.0001). Smokers had lower 25OHD level than the non-smokers (men p<0.0001; women p = 0.002). Use of hormone replacement therapy and absence of menstruation was associated with higher 25OHD level (p = 0.02 and 0.002, respectively). No association was seen between educational level and total household income with 25OHD level.

**Table 2:** 25OHD concentrations in the Finnish population aged 45–74 years.

	Men			Women		
Variable	Mean 25OHD (nmol/l) ± SD (n)	p-value	p-value (adjusted*)	Mean 25OHD (nmol/l) ± SD (n)	p-value	p-value (adjusted*)
All	58.2±16.9 (1348)			57.1±17.3 (1474)		
*Age*						
45–49 years	54.0±15.3 (193)	<0.0001	<0.0001	52.3±16.4 (221)	<0.0001	<0.0001
50–54 years	55.3±15.2 (198)			55.1±15.5 (261)		
55–59 years	58.5±17.8 (234)			58.2±18.7 (257)		
60–64 years	58.0±18.6 (215)			58.9±18.6 (247)		
65–69 years	61.7±16.9 (275)			58.3±16.4 (282)		
70–74 years	60.0±15.9 (233)			59.4±17.4 (206)		
*Month of sample collection*						
October	61.0±16.9 (559)	<0.0001	<0.0001	58.9±17.3 (687)	0.001	0.002
November	56.4±16.1 (532)			55.5±17.2 (543)		
December	56.0±17.7 (257)			55.6±17.2 (244)		
*BMI*						
<25 kg/m^2^	59.7±18.5 (389)	0.04	0.12	60.0±17.0 (518)	<0.0001	<0.0001
25–29.9	58.0±15.9 (664)			58.4±18.7 (553)		
30–34.9	57.6±17.4 (224)			52.8±14.8 (278)		
>35	53.9±14.4 (70)			48.7±13.1 (124)		
*Physical activity*						
Low	53.9±17.8 (267)	<0.0001	<0.0001	51.8±17.5 (270)	<0.0001	<0.0001
Moderate	58.3±15.9 (760)			57.5±16.9 (872)		
High	62.1±17.4 (295)			60.6±17.6 (307)		
*Use of vitamin D containing supplements*						
No	57.0±16.8 (1105)	<0.0001	<0.0001	53.9±16.7 (929)	<0.0001	<0.0001
Yes	63.9±16.1 (243)			62.5±17.1 (545)		
*Smoking*						
No	59.6±16.3 (1069)	<0.0001	0.0002	57.6±17.3 (1308)	0.002	0.03
Yes	53.2±18.3 (261)			52.9±17.8 (153)		
*Use of hormone replacement therapy*						
No	-	-	-	56.4±17.4 (1016)	0.02	0.48
Yes	-	-	-	58.9±17.2 (335)		
*Menstruation*						
No	-	-	-	58.0±17.7 (1118)	0.002	0.27
Yes	-	-	-	54.4±16.0 (304)		
*Educational level*						
0–9 years	58.2±16.8 (557)	0.32	0.93	57.0±16.6 (507)	0.97	0.63
10–12 years	57.4±17.3 (381)			57.2±16.8 (434)		
>13 years	59.2±16.5 (393)			57.2±18.6 (506)		
*Total household income*						
<30 000 €/year	57.3±16.5 (529)	0.16	0.28	57.5±17.5 (640)	0.34	0.51
30 001–60 000 €/year	59.3±16.9 (534)			56.5±17.0 (513)		
60 001–80 000 or more €/year	58.6±15.3 (211)			58.5±18.3 (204)		

*adjusted for age, gender, BMI, month of sample collection, physical activity and smoking.

Statistically significant association of 25OHD level with age (in men and women p<0.0001), month of sample collection (in men p<0.000; in women p = 0.002), physical activity (in men and in women p<0.0001), use of vitamin D supplements (in men and in women p<0.0001) and smoking (in men p = 0.0002; in women p = 0.03) remained in the multivariate model when adjusted for age, gender, BMI, month of sample collection, physical activity and smoking (table 2). The association of 25OHD with BMI remained significant only in women (p<0.0001). Use of hormone replacement therapy and presence of menstruation were not associated with 25OHD level in the multivariate model.

**Figure 1:**
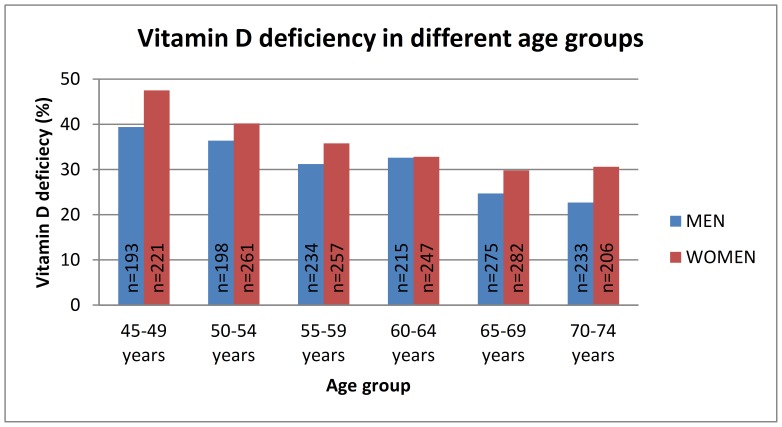
Prevalence of vitamin D deficiency (25OHD<50 nmol/l) in different age groups. P = 0.007 in men and p<0.0001 in women (adjusted for gender, BMI, month of sample collection, physical activity and smoking).

**Figure 2:**
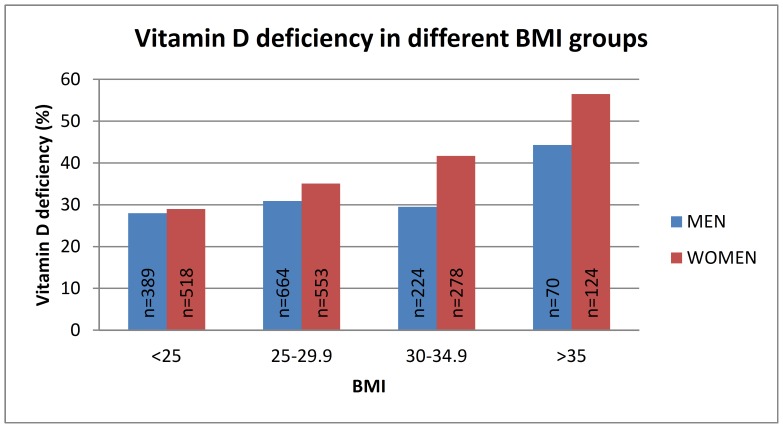
Prevalence of vitamin D deficiency (25OHD<50 nmol/l) in different BMI groups. P = 0.10 in men and p<0.0001 in women (adjusted for age, gender, month of sample collection, physical activity and smoking).

The proportion of men and women with 25OHD deficiency (<50 nmol/l), sufficiency (50–75 nmol/l) and with an optimal 25OHD level (>75 nmol/l) was calculated. Of men 30.6% had vitamin D deficiency, 54.0% a sufficient and 15.4% an optimal 25OHD level. Of women 36.0% had vitamin D deficiency, 49.5% a sufficient and 14.5% an optimal 25OHD level. Vitamin D deficiency was more common among women than men (p = 0.01). Only 1% (n = 13) of the men and 0.7% (n = 10) of the women had severe vitamin D deficiency (25OHD <25 nmol/l). Prevalence of vitamin D deficiency (25OHD <50 nmol/l) in different age and BMI groups is shown in figure 1 and 2.

### 25OHD level in relation to features of MetS and glucose regulation

The association of 25OHD level with MetS, components of MetS and with glucose regulation is presented in table 3. In the univariate analysis the presence of MetS was associated with lower 25OHD level in women (p = 0.0001). Higher waist circumference in women (p = 0.001) and higher triglyceride concentration both in men (p = 0.0007) and in women (p<0.0001) associated with low 25OHD level. 25OHD level was lower in those women that had hs-CRP concentration higher than 10 mg/l than those that had lower than 10 mg/l (p = 0.04).

**Table 3:** Association of 25OHD level with metabolic syndrome, type 2 diabetes and estimated cardiovascular disease risk (Framingham score) in the Finnish population aged 45–74 years.

	Men			Women		
	Mean 25OHD (nmol/l) (n)	p-value	p-value (adjusted*)	Mean 25OHD (nmol/l) (n)	p-value	p-value (adjusted*)
**Metabolic syndrome (ref. 17)**						
No	58.9±17.6 (540)	0.24	0.72	59.0±17.7 (675)	0.0001	0.15
Yes	57.8±16.4 (804)			55.4±16.9 (793)		
**Metabolic syndrome components**						
*Waist circumference*						
<94 cm (men); <80 cm (women);	59.0±18.6 (403)	0.34	0.59	59.9±16.8 (314)	0.001	0.82
>94 cm (men); >80 cm (women)	58.0±16.1 (942)			56.3±17.4 (1155)		
*HDL*						
>1.0 mmol/l (men); >1.3 mmol/l (women)	58.2±17.1 (1181)	0.86	0.44	57.5±17.7 (1130)	0.10	0.51
<1.0 mmol/l (men); <1.3 mmol/l (women)	58.4±15.4 (167)			55.7±16.1 (344)		
*Triglycerides*						
<1.7 mmol/l	59.1±16.9 (999)	0.0007	0.02**	58.2±17.6 (1203)	<0.0001	0.001**
>1.7 mmol/l	55.6±16.7 (349)			52.2±15.1 (271)		
*Blood pressure*						
Systolic<130 and/or diastolic<85 mm Hg	58.8±16.2 (341)	0.48	0.17	58.0±17.6 (458)	0.15	0.06
Systolic>130 and/or diastolic>85 mm Hg	58.1±17.1 (1006)			56.6±17.2 (1014)		
*Fasting glucose*						
<5.6 mmol/l	58.6±17.6 (164)	0.79	0.81	58.3±17.9 (451)	0.08	0.67
>5.6 mmol/l	58.2±16.8 (1184)			56.6±17.1 (1023)		
**Glucose regulation**						
Normal glucose regulation (NGR)	58.6±16.9 (479)	0.97	0.96	58.3±17.8 (813)	0.07	0.22
Type 2 diabetes (known and screen-detected together)	58.0±14.0 (104)			56.6±18.3 (71)		
Screen-detected diabetes mellitus (SDM)	57.5±18.4 (173)			55.8±16.2 (123)		
Impaired glucose tolerance (IGT)	58.5±16.2 (241)			55.2±16.1 (260)		
Impaired fasting glucose (IFG)	58.4±17.5 (337)			55.7±17.5 (188)		
**hs-CRP**						
<10 mg/l	58.3±16.8 (1314)	0.75	0.82	57.3±17.4 (1417)	0.04	0.93
>10 mg/l	57.3±20.2 (34)			52.5±15.9 (57)		

*adjusted for age, gender, BMI, month of sample collection, physical activity and smoking.

**adjusted for age, gender, BMI, month of sample collection, physical activity, smoking and waist circumference.

In the multivariate model (adjusted for age, gender, BMI, month of sample collection, physical activity, smoking and waist circumference) association of 25OHD level with triglyceride concentration remained statistically significant both in men (p = 0.02) and women (p = 0.001), whereas association with presence of MetS, waist circumference and hs-CRP in women did not. No association between 25OHD and HDL concentration, blood pressure, fasting glucose, type 2 diabetes or glucose regulation was seen in this study.

### 25OHD level in relation to CVD markers

**Figure 3:**
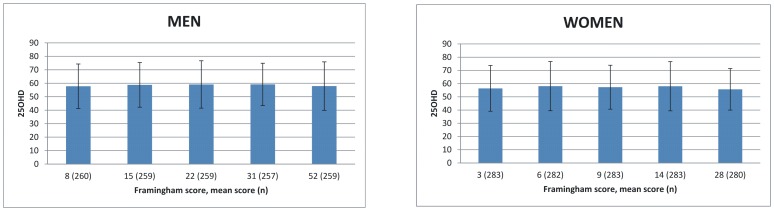
Association of 25OHD (nmol/l) with Framingham score. Mean Framingham risk score means the average risk score in each quintile of the study population. Higher score represents higher risk for CVD. P = 0.47 in men and p = 0.44 in women (adjusted for age, gender, BMI, month of sample collection, physical activity and smoking).

**Figure 4:**
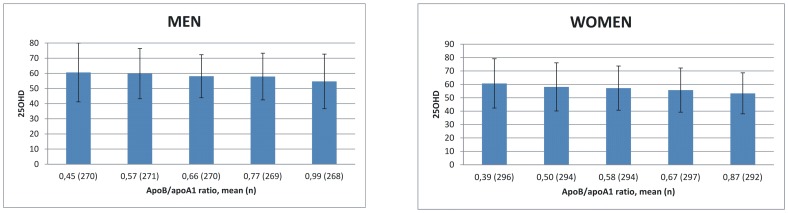
Association of 25OHD (nmol/l) with apoB/apoA1 ratio. Mean apoB/apoA1 ratio means the average ratio in each quintile of the study population. Higher ratio represents higher risk for CVD. P = 0.04 in men and p = 0.0002 in women (adjusted for age, gender, BMI, month of sample collection, physical activity, smoking and waist circumference).

The association of 25OHD level with CVD markers is shown in figure 3 and 4. No association was found between 25OHD and Framingham score in the univariate analysis (p = 0.67 in men and p = 0.44 in women) or when adjusted for age, gender, BMI, month of sample collection, physical activity and smoking (p = 0.47 in men and p = 0.44 in women). Higher apoB/apoA1 ratio associated with lower 25OHD level in the univariate analysis both in men (p = 0.0005) and in women (p<0.0001). Association remained statistically significant (p = 0.04 in men and p = 0.03 in women) when adjusted for age, gender, BMI, month of sample collection, physical activity, smoking and waist circumference.

## Discussion

In this population-based study representing a relatively large cohort, we analyzed association of 25OHD with several life-style factors and metabolic and cardiovascular disease markers. Our results are mainly consistent with a previous large (n = 5714) Finnish population-based study [27], where samples were collected during year 2000 (during year 2007 in the present study). The mean 25OHD level was higher in our study (58.2 nmol/l in men and 57.1 nmol/l in women) than in the previous study (45.5 nmol/l in men and 45.2 nmol/l in women). The difference may be explained by fortification of liquid dairy products with vitamin D since 2003 in Finland. Furthermore, better awareness of vitamin D deficiency has led to an increase in the use of vitamin D supplements; 10.2% of the participants used vitamin D supplements during year 2000, whereas in the present study 18% of men and 37% of women used vitamin D supplements. Methodological differences in 25OHD analysis in these two studies, however, may complicate reliable comparison of the mean 25OHD level between these two time points.

We found a positive association between 25OHD level and age in our study population aged 45–74 years. The result is somewhat unexpected since an inverse association between 25OHD and age has been shown in several previous large population-based studies [28]–[30], and since the elderly are thought to be at a special risk of vitamin D deficiency due to diminished outdoor activity, one-sided diet and a decline in the ability of the skin to synthesize vitamin D [28], [31]. However, our results are consistent with a study of Jääskeläinen et al. [27], where a positive association between 25OHD and age was seen in Finnish population aged 30–79 years. Among Finnish people, the average retirement age was 60 years when the study was done. People aged 60–74 years could thus be regarded as “active pensioners”, still quite healthy and spending time travelling abroad and in the home country. It is typical for Finnish pensioners to spend time at the summer cottages were outdoor living is usual. Eating fish is also more common among older than younger Finns [2]. It is thus possible that older Finns get more vitamin D from the diet and also from the sun by spending more time outdoors and by travelling, which is mirrored by higher 25OHD level of the older Finns all year round. However, since this study was restricted to people aged 74 years or less, and the study of Jääskeläinen et al. [27] to people aged 79 years or less, it cannot be determined if the positive association of 25OHD with age remains in the elderly aged 80 years or more.

In this study we found an inverse association between 25OHD level and BMI. Association of low 25OHD level and obesity has been seen in several studies [32]–[34]. It is likely that higher BMI leads to lower 25OHD level, whereas lower 25OHD is unlikely to have an effect on BMI [35]. It is not known whether low 25OHD level is due to for example changes in vitamin D metabolism in obese individuals or life style factors associated with obesity, for example low sun light exposure. It is known that 25OHD is stored in adipose tissue and muscle [36]. It is possible that bioavailability of 25OHD or its precursors stored in adipose tissue is poor [36]. Recently it has been suggested that muscle provides an extravascular pool through which 25OHD circulates [37], [38]. This could explain the positive association between physical activity and 25OHD level found in this study. Also previously, high 25OHD level have been positively associated with outdoor activity [27], [39], [40] and indoor exercise [41]. 25OHD level is known to associate with muscle strength and function [42], [43]. Low vitamin D status associates with poorer physical performance and a greater decline in physical performance and increases the risk of falls at least in the elderly [44], [45].

The present study confirms that smoking is an independent lifestyle factor associated with low 25OHD level both in men and women. Also previously it has been shown that low 25OHD level is associated with smoking [27], [46], [47] and smoking related cancers [48]. It is not known whether the inverse association between 25OHD level and smoking is due to life-style factors not accessed in this study (for example diet). It has been suggested though that chemicals in tobacco smoke may have a direct effect on vitamin D metabolism and function [46]. There is evidence that smoking alters expression of several genes, among which are some genes acting in the metabolic pathway of vitamin D [46], [49]. Low-grade chronic inflammation has been associated with low 25OHD level [50] and smoking increases inflammation [51]. There may be a benefit in increasing vitamin D supplementation among smokers, since the biologically active form of vitamin D controls inflammation by for example inhibiting the differentiation and maturation of dendritic cells to antigen presenting cells, and by down-regulating Th1 cell associated cytokine production [52]–[54].

Vitamin D status of Finnish adult population seems to have improved since the last population-based study from year 2000 [27], although still approximately one third of the participants were found vitamin D deficient. Especially women that have high BMI, low physical activity and that do not use vitamin D supplements are at increased risk of vitamin D deficiency. There has been an increase in recommended vitamin D supplementation for people aged 60 years or more in Finland after the sample collection of the present study, and thus it is likely that mean 25OHD level is higher at the moment.

Association of 25OHD with glucose regulation and type 2 diabetes has been shown in several studies [55] including studies done in Finland [56]. 25OHD level has been associated with insulin sensitivity indices [57], and low 25OHD has been shown to predict later development of type 2 diabetes [58]. In this study, however, no association between 25OHD and type 2 diabetes or glucose regulation was found. The inverse association between 25OHD and triglycerides found in the present study has consistently been shown in previous studies as well [59]. However, intervention studies have for the most part failed to show improvement in triglyceride level after vitamin D supplementation [60], [61] and thus a causal relationship has not been shown. In this study, 25OHD level was independently associated with apoB/apoA1-ratio. A positive association between apoA1 concentration and 25OHD level has been previously shown [62], [63]. Obesity associates with both high triglyceride concentration and high apoB/apoA1 ratio. Low grade inflammation is associated with obesity as well as with CVD [64] and thus it is possible that associations of 25OHD with triglyceride concentration and apoB/apoA ratio relate to the known connection of vitamin D with inflammation [50]. However, in this study 25OHD level was not associated with hs-CRP, which was the only inflammation marker analyzed in this population.

Non-participation is a source for possible biases in this study since in population-based studies typically participants are healthier than the non-participants [65]. However, this would mean that the associations found between 25OHD and disease markers as well as between 25OHD and lifestyle factors representing unhealthy lifestyle can be assumed to be stronger rather than weaker in the whole population and thus does not explain the associations found in the present study. Also, it should be noted that in international comparison there are not many surveys which include a personal health examination with 2-hour glucose tolerance test with participation rate as high as in this survey (65%).

In conclusion, several lifestyle and clinical factors are associated with 25OHD level. These confounding factors should be taken into account when analysing the association of 25OHD with the risk for different diseases. The findings of the study indicate that in the future molecular mechanisms behind the associations of 25OHD e.g. with physical activity and smoking should be examined. Also, potential benefit of increased vitamin D supplementation for smokers should be evaluated. The association of 25OHD with BMI, high triglyceride concentration and apoB/apoA1 ratio may be related to the role of vitamin D in inflammation, but more detailed studies are needed.

Note: A part of the results of this study have been previously published in Finnish: Miettinen M, Kinnunen L, Keinänen-Kiukaanniemi S, Korpi-Hyövälti E, Niskanen L, Oksa H, Saaristo T, Sundvall J, Tuomilehto J, Vanhala M, Uusitupa M, Peltonen M. Prevalence of vitamin D insufficiency in Finnish adult population. Suom Lääkäril. 2013;4:29–33. [in Finnish].
